# Analysis of Microbiome Data in the Presence of Excess Zeros

**DOI:** 10.3389/fmicb.2017.02114

**Published:** 2017-11-07

**Authors:** Abhishek Kaul, Siddhartha Mandal, Ori Davidov, Shyamal D. Peddada

**Affiliations:** ^1^Biostatistics and Computational Biology Branch, National Institute of Environmental Health Sciences (NIH), Durham, NC, United States; ^2^Public Health Foundation of India, Gurgaon, India; ^3^Department of Statistics, University of Haifa, Haifa, Israel

**Keywords:** Microbiome data, Aitchisons log-ratio, bootstrap, covariates, cross-sectional data, false discovery rate (FDR)

## Abstract

**Motivation:** An important feature of microbiome count data is the presence of a large number of zeros. A common strategy to handle these excess zeros is to add a small number called pseudo-count (e.g., 1). Other strategies include using various probability models to model the excess zero counts. Although adding a pseudo-count is simple and widely used, as demonstrated in this paper, it is not ideal. On the other hand, methods that model excess zeros using a probability model often make an implicit assumption that all zeros can be explained by a common probability models. As described in this article, this is not always recommended as there are potentially three types/sources of zeros in a microbiome data. The purpose of this paper is to develop a simple methodology to identify and accomodate three different types of zeros and to test hypotheses regarding the relative abundance of taxa in two or more experimental groups. Another major contribution of this paper is to perform constrained (directional or ordered) inference when there are more than two ordered experimental groups (e.g., subjects ordered by diet or age groups or environmental exposure groups). As far as we know this is the first paper that addresses such problems in the analysis of microbiome data.

**Results:** Using extensive simulation studies, we demonstrate that the proposed methodology not only controls the false discovery rate at a desired level of significance while competing well in terms of power with DESeq2, a popular procedure derived from RNASeq literature. As expected, the method using pseudo-counts tends to be very conservative and the classical t-test that ignores the underlying simplex structure in the data has an inflated FDR.

## 1. Introduction

Microbial count data are represented using operational taxonomic units (OTUs) from 16S rRNA studies. For each specimen (e.g. fecal sample) drawn from an ecosystem (e.g. gut), the number of occurrences of each OTU is measured and the resulting OTU table is summarized to obtain relative abundance for bacterial taxa in a specimen. These OTU counts may be summarized at any level of the bacterial phylogeny, e.g., species, genus, family, order, etc. Throughout this paper we use the generic term “taxa” to denote a particular phylogenetic classification. Since the relative abundances of taxa in a specimen sum to 1, these are compositional data and they reside in a simplex rather than the entire Euclidean space. Another important feature of these microbiome data is that not all taxa may be present in each sample, i.e., some of the OTUs may take zero values. Using such microbial compositional data, researchers are interested in understanding the interplay between microbiome, diet, genome and human health (Clemente et al., 2012; den Besten et al., 2013). Accordingly, there is an urgent need for statistical methods for analyzing these complex microbial count data. This is an active area of research and a variety of statistical and computational methods have been proposed in the literature to answer a variety of scientific questions. For a review one may refer to Li (2015) and Mandal et al. (2015). The latter described in detail various statistical parameters associated with microbial compositional data and discuss which are estimable, and hence testable, and which are not. They proposed Aitchison's log-ratio based methodology (Aitchison, 1982, 1985, 1986) called ANCOM for comparing the taxa abundance at the ecosystem level in two or more groups or populations. Earlier, Xia et al. (2013) also considered Aitchison's log-ratio based methodology for microbiome data and proposed a penalized likelihood based methodology to select covariates influencing microbiome expression.

Excess zeros in microbiome data present a challenge when analyzing these data, specifically when comparing two or more experimental groups. A common strategy to handle these excess zeros is to add a small number called pseudo-count (e.g., 1, cf. Xia et al., 2013; Mandal et al., 2015). Although adding a pseudo-count appears to be a reasonable and a simple strategy, it is *ad-hoc*. Other strategies include modeling excess zeros using various probability models (Paulson et al., 2013; Chen and Li, 2016). However, such models often make an implicit assumption that all zeros can be explained by a common probability model. As described in this article, this is not always the case as there are potentially three different sources of zeros in microbiome data. The first major contribution of this paper is a method which identifies the three major types or sources of zeros in microbiome data. The second major contribution of this paper is to compare the mean relative abundance of taxa in two or more groups while taking into consideration the compositional structure and the type of zeros in the data. Unlike ANCOM (Mandal et al., 2015), which compares the taxa abundance in the ecosystem of two or more groups, the proposed methodology compares the abundance of taxa relative to a background value. The method is general enough that the reference background value can be a specific taxon the user is interested in or it can be some suitable background value specific to each specimen, such as the geometric mean (Aitchison's centered log-ratios). The main idea is to normalize data within each specimen so that any background values within the specimen are eliminated. This idea is analogous to what is often done in gene expression studies. If a particular taxon is used as the reference taxon or reference value, then we assume that the taxon is present in all specimens. Thus the normalizing variable is same across all specimens. From our experience, in practice this condition is not particularly stringent, especially if the researcher is interested in studying microbiome at the genus or a higher level of the phylogenetic tree. For example, in the Yatsunenko et al. (2012) study consisting of 531 samples over three geographical locations (US, Venezuela and Malawi) there exist at least one taxon (at the genus level) that is present in all samples. These data are discussed later in this manuscript. If no such taxon exists, then the proposed methodology can be implemented using the geometric mean as the reference to correct for the background abundance levels of each specimen.

In some applications researchers are interested in performing inferences regarding mean relative abundances of individual taxon in the ecosystems of more than two ordered groups. For example, one may be interested in comparing the mean relative abundances of individual taxon in subjects ordered by different levels of fat intake or levels of dietary supplements or subjects belong to different age groups etc. In all such situations the classical two-sided tests are not as informative or powerful as the constrained inference (or order restrictions) based tests (Farnan et al., 2014; Jelsema and Peddada, 2016). Since the proposed methodology converts the simplex data to Euclidean space data, constrained inference theory developed in Farnan et al. (2014) is directly applicable to the present setting. Thus the third major contribution of this paper is to perform constrained inference when there are more than two ordered experimental groups. As far as we know this is the first paper that addresses such problems in the analysis of microbiome data. Owing to the generality of Farnan et. al. methodology to (a) cross-sectional as well as repeated measures/longitudinal designs, (b) detecting trends in the relative abundances of taxa in two or more ordered experimental groups such as in time course experiments, dose-response studies or when comparing subjects at stages of disease, (c) multiple pairwise comparisons of several experimental groups against a pre-specified control group, the methodology described in this paper is therefore very broadly applicable. Thus, the proposed methodology can be used for testing a wide range of hypotheses while controlling for false discovery rate (FDR) at the desired nominal level. Extensive simulations are performed to demonstrate that the proposed methodology controls the FDR in a variety settings considered in the simulation study while enjoying higher power than some commonly used methods including those based on pseudo-counts. We illustrate the methodology using the global gut data of Yatsunenko et al. (2012).

## 2. Notation and problem formulation

Suppose a sample of *n*_*j*_ specimens are drawn from the *j*^*th*^ experimental group, *j* = 1, 2, …, *J*. On each specimen suppose the abundance of *p* taxa are obtained. Here the word “taxa” could be at any level of the bacterial phylogeny, e.g., species, genus, family, order, etc., or just the counts of OTU categories themselves. Let *z*_*ijk*_ denote the observed abundance of *k*^*th*^ taxon, *k* = 1, 2, …, *p*, in the *i*^*th*^ specimen from the *j*^*th*^ experimental group. In vector notation we have *z*_*ij*_ = (*z*_*ij*1_, …, *z*_*ijp*_). For simplicity of exposition throughout this paper, we shall take *n*_*j*_ = *n*, *j* = 1, 2, …, *J* even though the methodology does not require the design to be balanced. As explained in Mandal et al. (2015), unlike most commonly encountered biological data, the basic counts of OTU categories within each specimen cannot be regarded as absolute values but only relative values as they depend upon the sampling depth corresponding to each specimen. In other words, it does not make sense to compare the expected value of the observed counts between two experimental groups. To draw any meaningful inferences regarding the taxa abundance in two or more groups one needs to “normalize” the data within each specimen. Since classical inference, such as t-tests or ANOVA are not valid in the present context due to the simplex constraint, following Aitchison (1980) and Mandal et al. (2015) worked with log-ratios of relative abundances within each specimen. This is equivalent to computing log-ratios of abundances of each taxon relative to a “reference value.” Thus, for the *i*^*th*^ specimen in the *j*^*th*^ experimental group, one may consider the following expression to normalize the data *z*_*ijk*_:

(2.1) $$\begin{array}{l} \text{log }z_{ijk}-f_{ij}\left(z_{ij1},\ldots ,z_{ijp}\right), \end{array}$$

using some pre-specified “reference value” *f*_*ij*_(*z*_*ij*1_, …, *z*_*ijp*_). For example, *f*_*ij*_(*z*_*ij*1_, …, *z*_*ijp*_) = log *z*_*ijb*_, where *z*_*ijb*_ is the count corresponding to a pre-specified reference taxon *b*. Alternatively, using the non-zero values *z*_*ijk*_, *k* = 1, 2, …, *p*, the user may choose $f_{ij}\left(z_{ij1},\ldots ,z_{ijp}\right)=r^{-1}\underset{\left\{k:z_{ijk}\neq 0\right\}}{\sum }\text{log }z_{ijk}$, where *r* is the number of non-zero components in ${\left(z_{ij1},z_{ij2},\ldots ,z_{ijp}\right)}^{\prime },$ i.e., the logarithm of the geometric mean of the OTU counts within each experimental group *j* = 1, …, *J* (Aittchisons centered log-ratio).

Although the above normalization procedure eliminates the effect of the library size within specimen, it does not account for differences in the library sizes across specimens. To deal with this, we make another correction to the above normalization step. We make the assumption that all specimens within an experimental group are a random sample from a common population of specimens so that the observed background value for a given specimen is a random realization from a common population of all background values. Thus we have the following one-way ANOVA model describing the observed background value:

(2.2) $$\begin{array}{l} f_{ij}\left(z_{ij1},\ldots ,z_{ijp}\right)=\mu_{j}+\varepsilon_{ij}, \end{array}$$

where μ_*j*_ is the fixed effect due to the experimental group *j* = 1, …, *J* and $\varepsilon_{ij}\sim N\left(0,\sigma_{\varepsilon }\right)$ is a random variable that captures variation due to the sampling depth. This quantity can then be predicted by the residual ${\hat{\varepsilon }}_{ij}=f_{ij}\left(z_{ij1},\ldots ,z_{ijp}\right)-\frac{1}{n}\underset{i\in j^{th}\text{ group}}{\sum }f_{ij}\left(z_{ij1},\ldots ,z_{ijp}\right)$ which can be interpreted as the best linear unbiased predictor (BLUP) in the assumed model.

Hence in place of the typical normalization (2.1), we normalize the raw abundances using the following normalized formula:

(2.3) $$\begin{array}{l} y_{ijk}=\text{log }z_{ijk}-\left(f_{ij}\left(z_{ij1},\ldots ,z_{ijp}\right)-{\hat{\mu }}_{j}\right) \end{array}$$

where ${\hat{\mu }}_{j}=\frac{1}{n}\underset{i\in j^{th}group}{\sum }f_{ij}\left(z_{ij1},\ldots ,z_{ijp}\right).$ This normalization procedure can be easily extended to the case when there are covariates present in the model. Of course, in the above formula, all logarithms are calculated under the assumption that there are no zero values. However, as mentioned earlier, this is not true with the microbiome data. We address this problem in the next section.

## 3. Zeros

A special feature of a microbiome data matrix is that it is higly sparse, i.e., a very high proportion of data entries are zero (absent taxa). For example, at the genera level, nearly 80% of the data matrix in the Global gut data of Yatsunenko et al. (2012) are zero. Furthermore, corresponding to a given taxon, the counts may vary from 0 to the order of 10^5^ across samples within an experimental group. In this section we develop a pre-processing step that not only helps us potentially understand the different types of zeros in the data but address them accordingly.

### 3.1. Outlier zeros

For a given taxon *k* in the *j*^*th*^ group, we declare the sample *i* to be an “outlier zero” if its count is zero and is declared to be an outlier by the methodology described below. In our assessment, this taxon is recorded as zero due to some extraneous reasons but not because it is below detection limits due to sampling depth. Thus, as far as taxon *k* is concerned, the *i*^*th*^ sample within group *j* is an outlier.

We first convert the count data into continuous scale by adding a pseudo-count of 1 and normalize the data using the transformation pseudo-count (2.3). Let *y*_*ij*_ = (*y*_*ij*1_, …, *y*_*ijp*_) denote the *p* dimensional vector for *i*^*th*^ observation in the *j*^*th*^ group, then for each *j, k*, we model *y*_*ijk*_ using the following mixture of normal distributions. Since our outlier detection algorithm is applied to each experimental group *j* and each taxon *k*, for simplicity of exposition, we drop the subscript *j* and *k* from the following:

(3.1) $$\begin{array}{l} y_{i}\sim^{i.i.d}\pi N\left(\mu_{1},\sigma_{1}\right)+\left(1-\pi \right)N\left(\mu_{2},\sigma_{2}\right),i=1,\ldots ,n \end{array}$$

The main idea of our methodology is that when means of the two normal distributions $N\left(\mu_{1},\sigma_{1}\right)$ and $N\left(\mu_{2},\sigma_{2}\right)$ in the above mixture are “well separated and the left cluster, i.e. cluster corresponding to mean μ_1_, forms only a small fraction of the total number of observations of the group, i.e. π is small, then it is reasonable to assume that the left cluster is a collection of outlier observations in the group and the observed zero might be a potential outlier. On the other hand, if the two groups are not well separated then the observed zero may not be an outlier zero but zero due to other reasons. Such zeros are handled later in this section.

**Identification of two clusters**: For a given taxon within a group, we declare that its distribution is a mixture of two “distant” normal distributions if the following two criteria are satisfied:

1. **Separation**: The 97.5th percentile of the first distribution does not overlap with the 2.5th percentile of the second distribution, i.e., μ_1_ + 1.96σ_1_ < μ_2_ − 1.96σ_2_.
2. **Frequency**: One distribution is “c % heavier” than other, i.e., π < *c* for some pre-specified *c*.

The above determinations, along with the estimation of parameters π, μ_1_, μ_2_, σ_1_, σ_2_ of the mixture (3.1) can be performed efficiently by an algorithm due to Peddada and Hwang (2002). We refer to the data cells identified by this mechanism as “outlier zeros” which are ignorable entries (replaced by NA in the data).

### 3.2. Structural zeros

In many cases, because of the nature of the experimental groups, some taxa are not supposed to be present in samples obtained from some groups but may be present in others. For example, babies exposed to antibiotics may be devoid of some taxa in their fecal samples, which are present in healthy babies not exposed to antibiotics. Although, in theory the antibiotics exposed babies are expected to be completely devoid to some taxa, due to variability in the exposure and other factors, such taxa may not be 100% missing in the antibiotics exposed babies. Suppose *p* represents the proportion of non-zero taxa across all specimens in an experimental group. Then we expect *p* to be close to zero, if not exactly zero, in experimental groups where the taxon is not expected to be present. We refer to such zeros as structural zeros. For the *j*^*th*^ taxon in the *k*^*th*^ experimental group, let ${\hat{p}}_{jk}=\sum_{i=1}^{n}z_{ijk}/n$. Then we declare the taxon to have a structural zero value if either of the following is true.

1. ${\hat{p}}_{jk}=0$
2. ${\hat{p}}_{jk}-1.96\sqrt{{\hat{p}}_{jk}\left(1-{\hat{p}}_{jk}\right)/n}\leq 0.$

Taxa that are identified as structural zeros in any given group are ignored from all future analyses for that group. Thus, for example, if in a study there are three experimental groups and if a particular taxon *t* is declared to have structural zero in Group 1 but not in Groups 2 and 3, then we automatically declare that taxon *t* is differentially abundant in Group 2 relative to Group 1 as well as in Group 3 relative to Group 1. We then compare the relative abundance of *t* between Groups 2 and 3 using the methodology developed in this paper.

### 3.3. Sampling zeros

If an observed zero in the data does not qualify as an outlier zero or as a structural zero, then we declare such a zero to be sampling zero, perhaps caused by the sampling depth. In other words, these zeros are potentially due to the fact the taxon is relatively a rare taxon compared to other taxa in the specimen and due to technological (or other) reasons it was not observed. These sampling zeros are imputed by using a small pseudo-count value (e.g., 1) before analyzing the data. More generally, an imputation approach could also be applied to these left over zeros, however this is outside the scope of this manuscript.

To summarize, using the above process, we obtain a modified data set where; (a) samples with structural zeros are suitably removed from the data matrix, (b) the outlier zeros are treated as missing at random (MAR) in the sense of Rubin (1976) and the corresponding entries are replaced as “NA”, and (c) the sampling zeros are imputed as 1.

## 4. Analysis of two or more groups

In rest of this paper, we work with normalized data *y* described in Equation (2.3) after suitably dealing with zeros as described in the previous section. For the *k*^*th*^ taxon in the *j*^*th*^ experimental group, for *i* = 1, 2…, *n*, let μ_*jk*_ = *E*(*y*_*ijk*)_ and $\sigma_{jk}^{2}=Var\left(y_{ijk}\right)$. Using the zeros corrected data, we obtain the following unconstrained estimators for μ_*jk*_ and $\sigma_{jk}^{2}$, for *j* = 1, 2, …, *J* and *k* = 1, 2, …, *p*:

(4.1) $$\begin{array}{l} {\hat{\mu }}_{jk}=\frac{\sum_{i=1}^{n}1\left[y_{ijk}\neq NA\right]y_{ijk}}{\sum_{i=1}^{n}1\left[y_{ijk}\neq NA\right]},\\ {\hat{\sigma }}_{jk}^{2}=\frac{\sum_{i=1}^{n}1\left[y_{ijk}\neq NA\right]{\left(y_{ijk}-\hat{\mu }\right)}^{2}}{\sum_{i=1}^{n}1\left[y_{ijk}\neq NA\right]-1}\; \end{array}$$

In many applications, researchers are interested in comparing taxa relative abundances in two or more experimental groups. Depending upon the scientific question, one may perform a wide range of analyses. In this section we describe four different classes of analyses one may perform. In each case the statistical parameters of interest are μ_*jk*_, *j* = 1, 2, …, *J*, *k* = 1, 2, …, *p*. Note that, by construction, within each group *j*, $\overset{p}{\underset{k=1}{\sum }}\mu_{jk}=0$. Hence without loss of generality, we limit rest of the discussion to the first *p* − 1 taxa because $\mu_{jp}=-\overset{p-1}{\underset{k=1}{\sum }}\mu_{jk}$.

### 4.1. *H*_1_: two-sided global hypotheses

Since the data *y*_*ijk*_ belong to the Euclidean space, therefore for each taxon *k*, *k* = 1, 2, …, *p* − 1, we can use standard linear model based methodology to test such hypotheses on the group means μ_1*k*_, μ_2*k*_, …, μ_*Gk*_. adjusting for any covariates present in the data. If there are repeated measures or longitudinal data, then one can invoke the standard linear mixed effects models theory and test two-sided global hypotheses such as:

$$H_{0}:\mu_{1k}=\mu_{2k}=\ldots =\mu_{Jk}$$

Vs.

$$\mu_{rk}\neq \mu_{sk},$$

for some *r* ≠ *s*. The *p*-values obtained for each taxon *k*, *k* = 1, 2, …, *p* − 1, can be corrected for multiple testing using a suitable multiple testing correction procedure, such as Bonferroni or Benjamini-Hochberg (BH), depending upon the criterion of interest, namely, the Familywise error rate (FWER) or the false discovery rate (FDR).

### 4.2. *H*_2_: directional multiple pairwise testing

For each taxon *k*, *k* = 1, 2, …, *p* − 1, often researchers are not interested in testing the global hypotheses H_1_ but are interested in pairwise comparisons among some (or all) pre-specified experimental groups. Furthermore, within each pairwise comparison, a researcher may be interested in knowing if the (relative) abundance of a taxon increased or decreased from one group to the other. For example, a researcher may be interested in testing whether there is a greater (relative) abundance of *Bifidobacterium Sp*. in vaginally born babies who were never exposed to antibiotics during the first four months of life, than vaginally born babies who received at least one dose of antibiotics during the first four months. To draw such directional inferences in pairwise comparisons while controlling for the overall false discovery rate, one may apply the mdFDR (mixed directional FDR) controlling procedure introduced in Guo et al. (2010). When there are no covariates present, the Guo et al. (2010) procedure is available in the software ORIOGEN 4.1. https://www.niehs.nih.gov/research/atniehs/labs/bb/staff/peddada/.

### 4.3. *H*_3_: directional multiple pairwise testing against a specific experimental group

Hypotheses H_2_ deals pairwise comparisons among some pre-specified subset (or all) experimental groups. However, there are instances where researchers may be interested in testing all experimental groups against one pre-specified experimental group, such as, for example the control group. In such cases the power of Guo et al. (2010) procedure can be improved by appealing to the Dunnett's type test derived in Grandhi et al. (2016). The R-code for the method is provided in Grandhi et al. (2016).

### 4.4. *H*_4_: testing for patterns

In some applications, a researcher may not be specifically interested in pairwise comparisons, but may be interested in detecting overall trends/patterns in the relative abundance of a taxon over multiple ordered (or partially ordered) experimental groups. Order (or partial order) among experimental groups arises when the experimental groups represent time or dose or stages of disease etc.

For example, researchers may be interested in understanding the trends in (relative) abundance of taxa across four partially ordered groups, namely, (G1) Vaginally born babies who were not exposed to any antibiotics during the first four months after birth, (G2) Vaginally born babies who were exposed to at least one dose of antibiotics during the first four months of after birth, (G3) C-Section born babies who were not exposed to any antibiotics during the first four months after birth and (G4) C-Section born babies who were exposed to at least one dose of antibiotics during the first four months of after birth. In this case, groups G1 and G4 are the extreme groups in terms of gut microbial environment. In G1 there are no interventions, and in G4 there are two interventions (C-section and antibiotics exposure). Groups G2 and G3 are intermediate groups with one intervention each (either C-Section or antibiotics exposure). Although, groups G2 and G3 are intermediate to G1 and G4, the order between G2 and G3 is uncertain and hence we have a partial ordering among the four groups.

**Figure 1:**
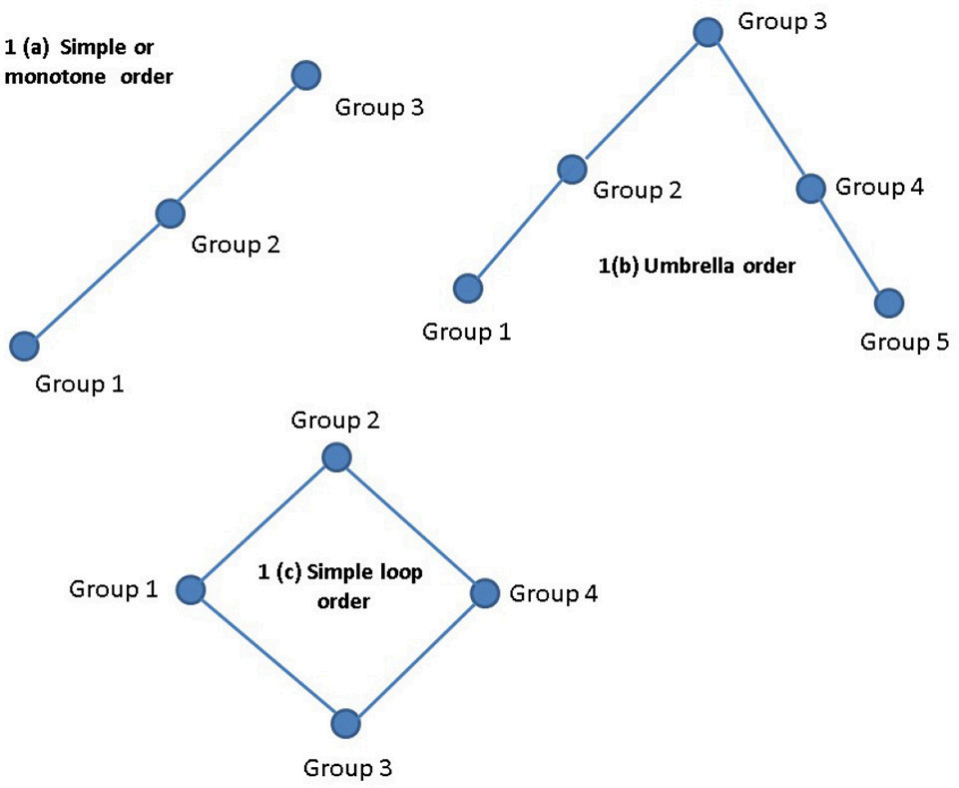
Illustration of hypotheses *H*_1*a*_ and *H*_2*a*_ testing for trends amongst groups.

A study design such as the one in this example can be represented using the Figure 1C, called a simple loop order, where, for each taxon, the researcher is interested in obtaining two sets of patterns, namely, pattern over G1, G2, and G4 and a pattern over G1, G3, and G4. Note that members within each set are completely ordered in terms of baby's exposure to interventions. When groups are ordered, one may be interested in identifying taxa whose mean relative abundance increases (or decreases) as we go from one extreme group (e.g., Group 1) to the other extreme group (e.g., Group 4) within each set. Such monotonic patterns, increasing or decreasing, are called the simple order (Figure 1A). More, precisely, for each taxon, *k* = 1, 2, …, *p* − 1, one may be interested in testing the following hypotheses:

$$H_{10}:\mu_{1k}=\mu_{2k}=\mu_{4k}$$

Vs.

$$H_{1a}:\left\{\mu_{1k}\leq \mu_{2k}\leq \mu_{4k}\right\}⋃\left\{\mu_{1k}\geq \mu_{2k}\geq \mu_{4k}\right\},$$

and

$$H_{20}:\mu_{1k}=\mu_{3k}=\mu_{4k}$$

Vs.

$$H_{2a}:\left\{\mu_{1k}\leq \mu_{3k}\leq \mu_{4k}\right\}⋃\left\{\mu_{1k}\geq \mu_{3k}\geq \mu_{4k}\right\}.$$

In some applications one may be interested in identifying taxa that have an umbrella shaped pattern as in Figure 1B.

As observed above, rather than using some arbitrary parametric functions, one can describe various patterns or trends using mathematical inequalities, called order restrictions. To determine the best pattern or trend for each taxon we adopt the strategy in Peddada et al. (2003), where a similar problem was considered for time-course gene expression data. For each taxon, we test the null hypothesis that there is no change in mean relative abundance (in log scale) over all the experimental groups against the alternative hypothesis which is the union of all patterns of interest. For each pattern we construct a suitable order restricted test and the final test statistic is taken to be the maximum of all test statistics. The null distribution of the test statistic is derived using the residual bootstrap based procedure developed in Farnan et al. (2014) which is implemented in the package called constrained linear mixed effects (CLME), an R code developed by Casey Jelsema and is described in Jelsema and Peddada (2016). The R code allows for modeling covariates as well as longitudinal/repeated measurements data. Since there are a large number of taxa, we perform multiple testing corrections using the BH procedure to control for the overall FDR. As in Peddada et al. (2003), if for a taxon, the null hypothesis is rejected at the desired level of significance (FDR ≤ α), then we assign the pattern with largest value of the test statistic. Thus, we are essentially adopting the ORIOGEN methodology developed in Peddada et al. (2003) to the present context.

## 5. Numerical results

We evaluate the performance of our proposed methodology, which we refer to as ANCOM-II, using two distinct simulation studies. The first is inspired by a real data set collected by Yatsunenko et al. (2012). This setup also allows for all three kinds of zeros described in the paper. The second is based on a negative binomial distribution, which is commonly used to model OTU count data of microbiome studies. The results of the proposed method are obtained by filtering outlier zeros at a threshold of *c* = 0.15. We compare the proposed with methodology with three other methods, namely, DESeq2 (Love, Huber and Anders 2014), t-test based on sample proportions (Prop-T) and t-test based on data transformed via (2.3) after adding a pseudo-count of 1 to each entry (Pseudo-C). Note that a comparison between ANCOM-II and the Pseudo-C method provides numerical results on how our assessment of zeros impacts the analysis. We also provide a user friendly R code in the supplementary materials to implement the proposed methodology described in this section.

### 5.1. Simulation study based on real data

This simulation study is based on the OTU count data (at the genus level) corresponding to the US group provided in Yatsunenko et al. (2012). We constructed two groups, namely, cases and controls (*J* = 2). Each group consisting of 175 subjects and 200 taxa. Among these 200 taxa, 100 are taken to be differentially abundant. As detailed below, our simulation study allows for all three forms of zeros discussed in the paper.

**Step 1** Generate a simple random sample of 175 subjects from the US group in Yatsunenko et al. (2012) data. Process the data as described in Section 2 by taking the genus *Bifidobacterium* as the reference taxon for the transformation (2.3). This provides us with a 175 × 661 data matrix. Let *m* = (*m*_1_, …, *m*_200_) denote the vector of 200 column means which are highest in magnitude obtained after normalization of (2.3).

**Step 2 (Outlier zeros)** Using the vector *m* simulate 175 case and control samples using a bimodal distribution as follows. For *i* = 1, .., 175

$$\begin{array}{l} y_{i1k}\sim^{iid}\pi N\left(m_{k}-3,1\right)+\left(1-\pi \right)N\left(m_{k}+3,1\right),\\ k=1,\ldots ,100\\ y_{i2k}\sim^{iid}\pi N\left(m_{k}-3,1\right)+\left(1-\pi \right)N\left(m_{k}+3,1\right),\\ k=1,\ldots ,50\\ y_{i2k}\sim^{iid}\pi N\left(m_{k}-3,1\right)+\left(1-\pi \right)N\left(m_{k}+3+\delta ,1\right),\\ k=51,..,100. \end{array}$$

For each simulated repetition π is chosen uniformly between (0.85,0.95).

**Step 3 (Sampling zeros)** Using the vector *m* simulate 175 case and control samples with a unimodal distribution. For *i* = 1, .., 175

$$\begin{array}{l} y_{i1k}\sim^{iid}N\left(m_{k},1\right),k=101,\ldots ,175\\ y_{i2k}\sim^{iid}N\left(m_{k},1\right),k=101,\ldots ,125\\ y_{i2k}\sim^{iid}N\left(m_{k}+\delta ,1\right),k=126,\ldots ,175 \end{array}$$

**Step 4 (Structural zeros)** Create 175 case and control samples for taxa that are structurally zero in the control group. For *i* = 1, .., 175, *k* = 176, …, 200, set *y*_*i*1*k*_ = 0 and $y_{i2k}=N\left(m_{k},1\right)$ with probability 0.01.

**Step 5** Back transform the above continuous scale data to the count scale by inverting the transformation (2.3) and rounding the observations. Specifically, using the transformation

$$\begin{array}{l} z_{ijk}=e^{y_{ijk}}\left[z_{ijb}/{\left(\prod_{i}z_{ijb}\right)}^{1/n}\right] \end{array}$$

here *z*_*ijb*_ represents the counts of “*Bifidobacterium*” taxa in the subset of the global gut data described in **Step 1**. In the above steps, all values between (0,1) are rounded to zero counts. Thus, although we are generating continuous random variables, with a positive probability we generate zeros. Recall that in **Step 2** samples are generated from a mixture of two independent normal distributions. The observations corresponding to zero counts are induced by the first component of the mixture distribution. Since the two components are independently generated, the zero observations are not dependent on the taxa itself (assuming that the true distribution of the taxa is given by the second component). Thus, these zeros, by design, represent observations that are missing at random. On the other hand, the zeros obtained in **Step 3** are from a single distribution, and are zero because *z*_*ijk*_ with values between 0 and 1 are set to 0.

**Step 6** Apply the three methods on the above simulated count data. Repeat Steps 1 through 6 and estimate the false discovery rate (FDR) and power of each method.

**Figure 2:**
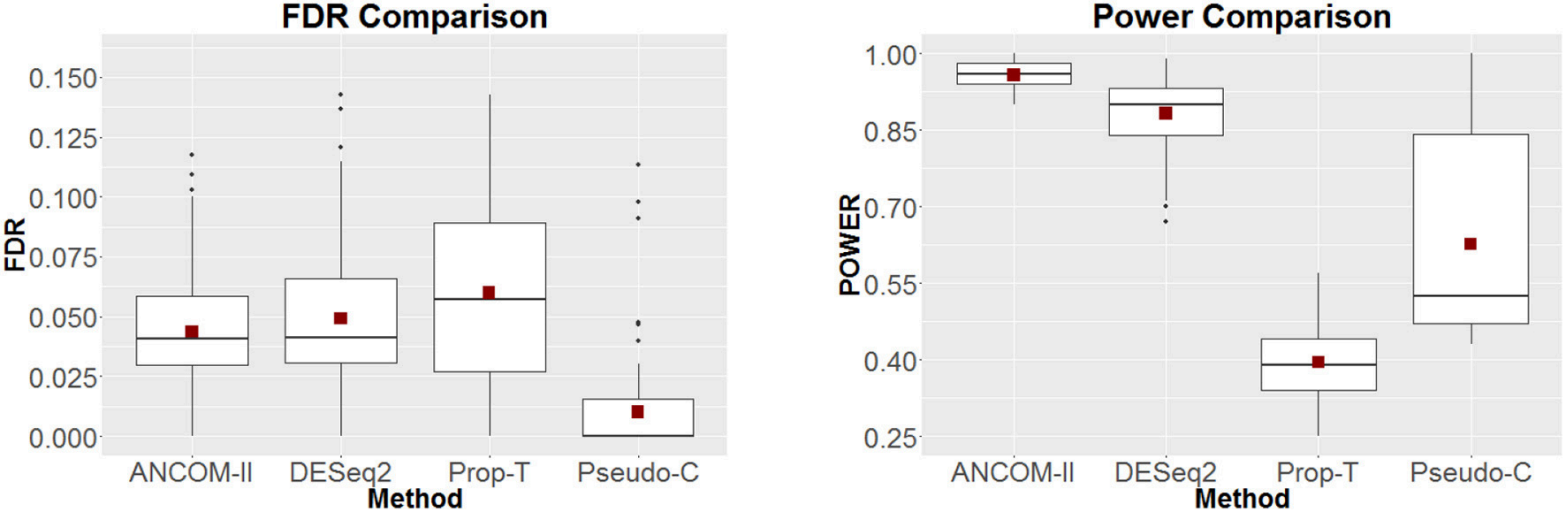
FDR **(Left)** and Power **(Right)** comparisons among ANCOM II, DESeq2, Prop-T, and Pseudo-C. Power comparisons are for δ = 0.5.

**Figure 3:**
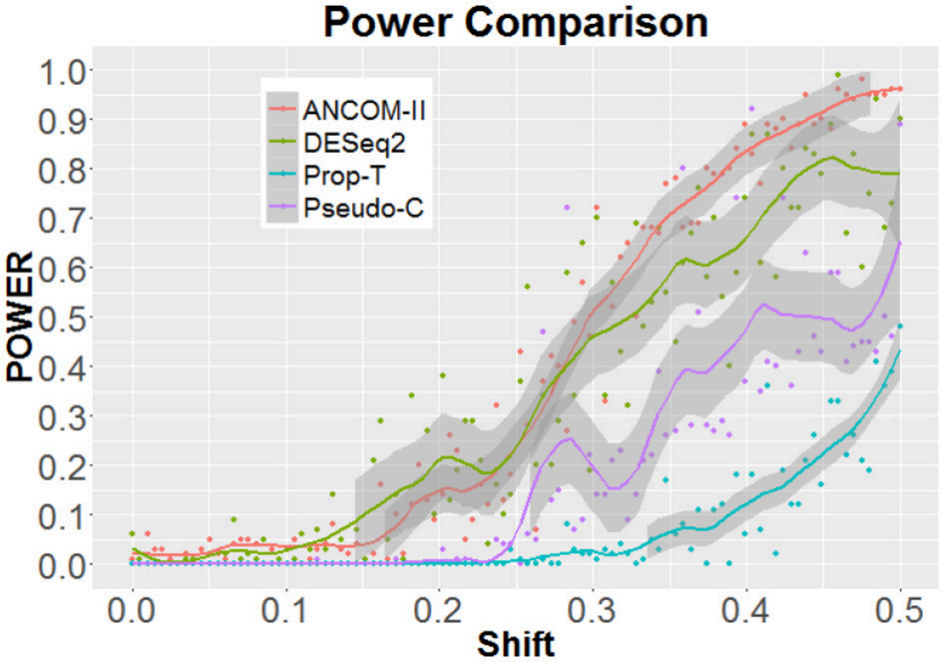
Power comparisons among ANCOM II, DESeq2, Prop-T, and Pseudo-C, for different values of δ ∈ (0, 0.5).

The left and right panels of Figure 2 provides the estimated FDR and power of the four methods, respectively. Here the shift parameter of Steps 2 and 3 is set to δ = 0.5. In this setting, on average (red dot), our proposed method, DESeq2 and Pseudo-C appear to control the FDR at the nominal level of 0.05. However, in terms of power our method appears to outperform the rest. In Figure 3, we further examine the effect of a varying shift parameter δ. We compare the powers of the four methods for 100 distinct values of δ ∈ (0, 0.5). Once again we note that the proposed method ANCOM-II, tends to have larger power than the others. Specifically, a comparison between ANCOM-II and the Pseudo-C method emphasizes the importance of identifying the various sources of zeros and dealing with them accordingly, rather than using a constant pseudo-count for all observed zeros.

### 5.2. Simulation based on negative binomial distribution

**Figure 4:**
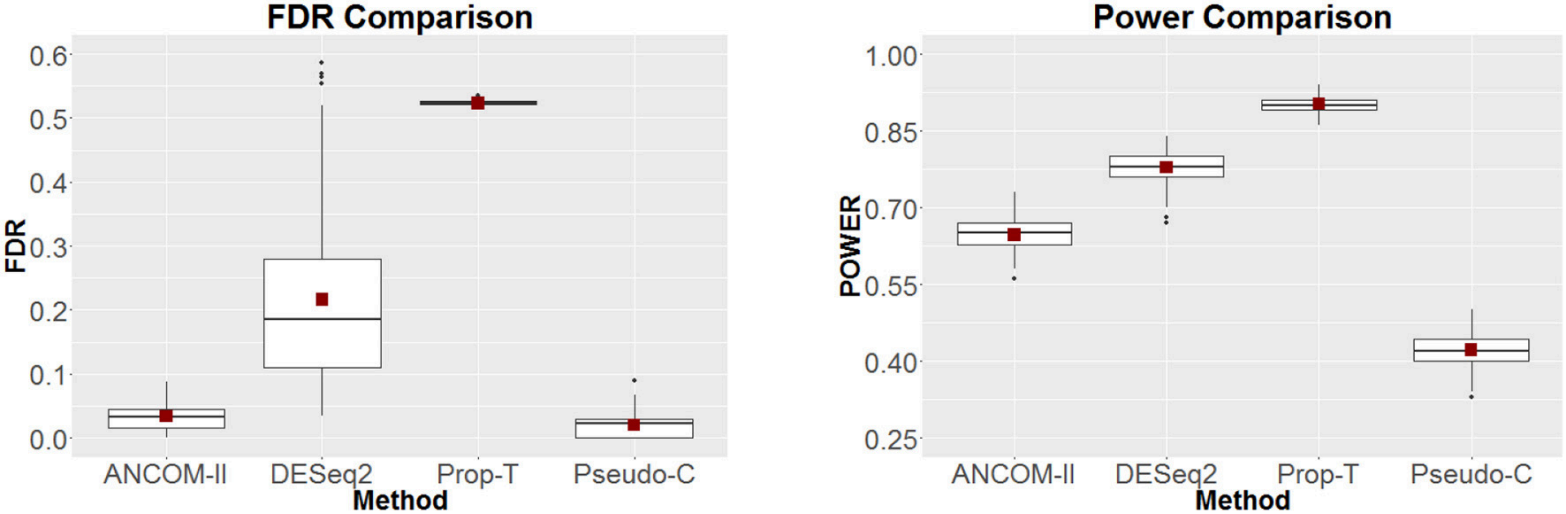
FDR **(Left)** and Power **(Right)** comparisons among ANCOM II, DESeq2, Prop-T, and Pseudo-C for simulation based on negative binomial distribution.

In this section we investigate the performance of the four methods by generating data according to negative binomial (NB) distribution as follows. For *j* = 1, 2, *k* = 1, …, 200, we generate,

(5.1) $$\begin{array}{l} z_{ijk}\sim NB\left(\mu_{jk},s_{jk}\right),\;i=1,..,100 \end{array}$$

where μ_*jk*_, *s*_*jk*_ are the mean and dispersion parameters of the negative binomial distribution respectively, in all cases we set $s_{jk}=\mu_{jk}^{2}.$ The control samples are generated for *j* = 1 and *k* = 1, .., 200 by choosing μ_*jk*_ from a uniform distribution over (1,1500). The case samples are generated by shifting the mean of the first one hundred taxa. Thus, for *j* = 2, *k* = 1, .., 100 set μ_*jk*_ = μ_1*k*_ + 5*k*. The remaining *k* = 101, …, 200 micorbes for group *j* = 2 are generated with the same mean parameters as the control samples. Furthermore we induce additional zeros in the data set by multiplying the previously generated counts with independent Bernoulli random variables *w*_*ijk*_ = 0 with probability 1−π_*jk*_ where π_*jk*_ is chosen uniformly between (0.8,1). This simulation experiment is repeated 100 times and the FDR and power comparison results are reported in Figure 4. From these simulation results we note that only ANCOM-II and Pseudo-C have estimated FDR at or below the nominal level of 0.05. Furthermore, between the two methods, ANCOM-II enjoys higher power. DESeq2 and Prop-T have unacceptably high estimated FDR.

## 6. Analysis of global human gut microbiome data

We illustrate ANCOM-II using global human gut microbiome data of Yatsunenko et al. (2012). The data consists of microbial taxa counts obtained from 317 subjects from US, 99 from Venezuela and 114 from Malawi. We used *Bifidobacterium* as the reference taxon because it was present in all samples.

Let *S*_*i*_ denote the set of genera with *i* countries having structural zeros. According to our method, by taking *c* = 0.15 we found that out of 661 genera, 262 belong to *S*_0_, 86 belong to *S*_1_, 95 belong to *S*_2_ and 218 belong to *S*_3_. Depending upon the set a genus belongs to, the method tests suitable hypotheses as outlined below (the corresponding R code is provided in the supplementary materials).

**Hypotheses 1**. For genera *j* ∈ *S*_0_ we test the following hypothesis

$$\begin{array}{l} H_{0j}:\mu_{US,j}=\mu_{Venezuela,j}=\mu_{Malawi,j},\text{ against}\\ H_{aj}:\{\mu_{US,j}\leq \mu_{Venezuela,j}\leq \mu_{Malawi,j}\}\\ \;\cup \{\mu_{US,j}\leq \mu_{Venezuela,j}\geq \mu_{Malawi,j}\}\\ \;\cup \{\mu_{US,j}\geq \mu_{Venezuela,j}\leq \mu_{Malawi,j}\}\\ \;\cup \{\mu_{US,j}\geq \mu_{Venezuela,j}\geq \mu_{Malawi,j}\} \end{array}$$

**Hypotheses 2a**. For genera *j* ∈ *S*_1_, when a taxon is structurally zero in Malawi data we test the following hypothesis

$$\begin{array}{l} H_{0j}:\mu_{US,j}=\mu_{Venezuela,j}\text{ against}\\ H_{aj}:\{\mu_{US,j}\leq \mu_{Venezuela,j}\}\\ \;\cup \{\mu_{US,j}\geq \mu_{Venezuela,j}\} \end{array}$$

**Hypotheses 2b**. For genera *j* ∈ *S*_1_, when a taxon is structurally zero in Venezuela data we test the following hypothesis

$$\begin{array}{l} H_{0j}:\mu_{US,j}=\mu_{Malawi,j}\text{ against}\\ H_{aj}:\{\mu_{US,j}\leq \mu_{Malawi,j}\}\\ \;\cup \{\mu_{US,j}\geq \mu_{Malawi,j}\} \end{array}$$

**Hypotheses 2c**. For genera *j* ∈ *S*_1_, when a taxon is structurally zero in US data we test the following hypothesis

$$\begin{array}{l} H_{0j}:\mu_{Venezuela,j}=\mu_{Malawi,j}\text{ against}\\ H_{aj}:\{\mu_{Venezuela,j}\leq \mu_{Malawi,j}\}\\ \;\cup \{\mu_{Venezuela,j}\geq \mu_{Malawi,j}\} \end{array}$$

**Hypotheses 3**. For genera *j* ∈ *S*_2_, which is structurally zero in Malawi and Venezuela data, we declare it to be differentially abundant (relative to a reference taxon) in the US compared to the other two countries. A similar conclusion is arrived for the other two possibilities.

**Hypotheses 4**. All genera belonging to thisset are discarded because they are considered to be absent in all three data sets.

**Figure 5:**
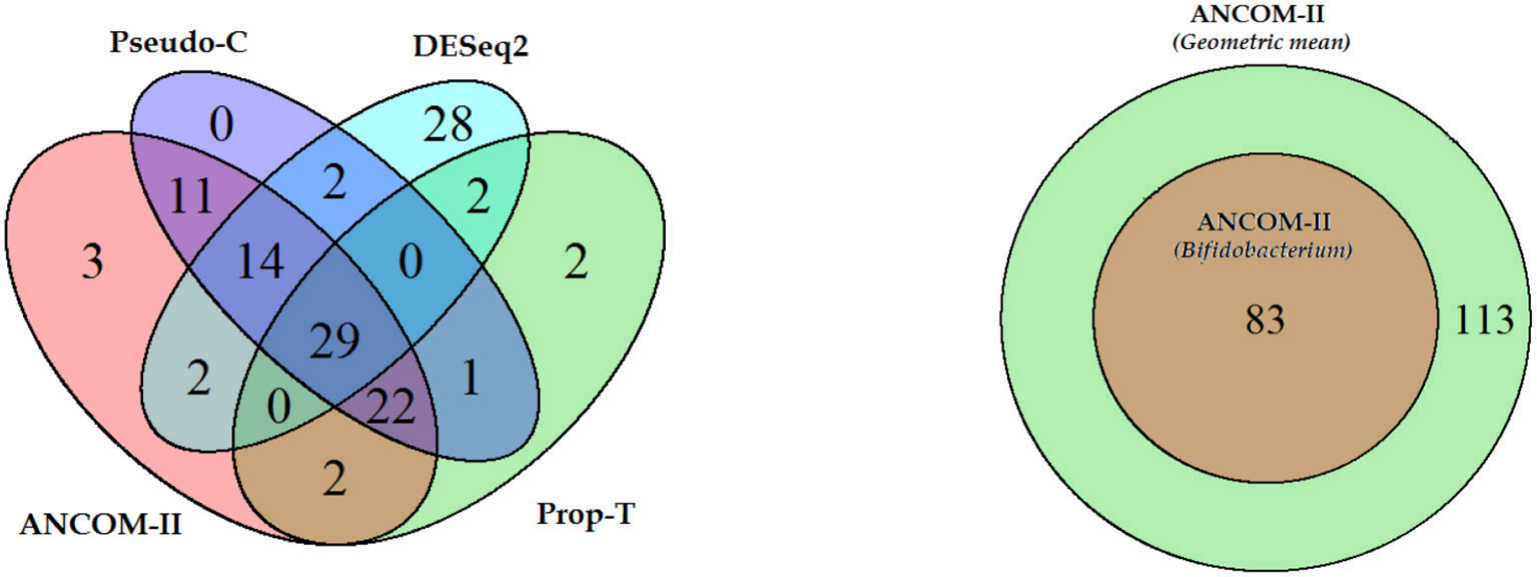
**(Left)** Venn diagram illustrating overlapping features detected by different procedures. **(Right)** overlapping features detected by assuming *Bifidobacterium* as normalizer or the geometric mean of all taxa as the normalizer.

Using the above approach ANCOM-II, relative to *Bifidobacterium* identified a total of 83 differentially abundant genera. Furthermore, ANCOM-II identified patterns of relative abundance of genera over the three countries. For genera in set *S*_0_ that are significant we discovered 34 genera belong to the phylum Firmicutes, followed by Proteobacteria (25), Actinobacteria (6), Tenericutes (5), Bacteroidetes (5) and others. Only 1 genera in set *S*_1_ (absent in Malawi) was found significant and belonged to the phylum Proteobacteria. Numbers within parenthesis represent the number genera within each phylum that were significant. We note that, the second highest number of differentially abundant genera belonged to phyla Proteobacteria. This is surprising given that this is typically one of the smaller phyla in the gut microbiome. This phylum consists of a large number of opportunistic pathogenic bacteria and an increased abundance of Proteobacteria is known to be associated with the disease necrotizing enterocolitis (NEC) Wang et al. (2009); Mai et al. (2011) and Inflammatory Bowel Disease (IBD), [Balfour Sartor and Mazmanian (2012)]. The genera in this phylum were observed to be uniformly lower in the US group as compared to the other two. A total of 29 taxa were present in US but structurally zero in Venezuela and Malawi, 53 were present in Venezuela but structurally zero in US and Malawi, lastly 13 were present in Malawi but structurally zero in Venezuela and US. In addition to ANCOM-II, we also applied DESeq2, Prop-T and Pseudo - C methods to these data. The results are summarized in the Venn diagram provided in Figure 5.

For comparison purposes, we re-analyzed the data using ANCOM-II but using the geometric mean (GM) of all non-zero taxa within subject as the reference, instead of *Bifidobacterium*. All taxa identified using *Bifidobacterium* as the reference taxon were a subset of taxa identified by the geometric mean as the reference taxon. The results are summarized in the Venn diagram in Figure 5.

## 7. Discussion

One of the challenges when dealing with compositional microbiome data is the presence of a large frequency of zero counts. At the moment there is no generally applicable methodology for comparing relative abundances of taxa among two or more populations/groups in presence of excess zero counts. In this article we took the first step toward identifying different types of zero counts and provided a strategy to deal with them. We take a principled approach to these data by classifying these zero counts into three different types. Inspired by gene expression studies, we proposed a simple method to “normalize” the data to eliminate specimen level effects. To deal with specimen specific background value, one may use a taxon that is present in all specimens, such as *Bifidobacterium* in the example considered in this paper, or one can use the geometric mean of taxa within the specimen. From our empirical studies, the choice of the background does not seem to affect the FDR, but could impact the power. Using this framework, a variety of statistical tests can be carried over from the literature depending upon the scientific question and hypotheses of interest. In this paper we describe four different types of statistical tests that are of common interest. Methodology developed in this paper, called ANCOM - II, is a general procedure that is not only applicable to cross-sectional as well as longitudinal designs, but in each case it can be used for detecting trends and patterns in a taxon over two or more groups. Our simulation study suggests that the methodology controls the overall false discovery rate while maintaining high power. In addition, since the methodology is based on residual bootstrap, it does not make any major distributional assumptions. For testing non-directional alternative hypotheses (hypothesis *H*_1_), ANCOM-II can be implemented using the R-code accompanying this paper. If no covariates are present and if there are no repeated measurements, then using residuals calculated in Equation (2.2) ANCOM-II can be implemented for testing directional alternatives *H*_2_, *H*_3_ by applying ORIOGEN. However, if covariates are present and if there are repeated measurements then ANCOM-II can be implemented for testing directional alternatives *H*_2_, *H*_3_ by applying CLME. At the moment we do not have a unified user friendly code that would be suitable for all scenarios described above. A general purpose software is being developed and we hope to release it in the near future.

## Author contributions

AK: Conceived the ideas, developed methodology, performed all numerical work and edited the manuscript. SM: Conceived the ideas and edited the manuscript. OD: Conceived the ideas and edited the manuscript. SP: Conceived the ideas, developed methodology and edited the manuscript.

### Conflict of interest statement

The authors declare that the research was conducted in the absence of any commercial or financial relationships that could be construed as a potential conflict of interest.
