# Supplementary material for: Analysis of Microbiome Data in the Presence of Excess Zeros
Source: Front Microbiol. 2017 Nov 7;8:2114. doi: 10.3389/fmicb.2017.02114 (PMC5682008; doi:10.3389/fmicb.2017.02114)
Supplement: Supplementary file 2 [file Presentation1.PDF]

Implementation Software: Analysis of Microbiome Data in the Presence of Excess Zeros

Abhishek Kaul, Siddhartha Mandal, Ori Davidov and Shyamal D Peddada

Implementation

This document provides details on implementing the methodology proposed in the manuscript "Analysis of Microbiome Data in the Presence of Excess Zeros." A sample data set has also been included in the documentation. The main function that performs this analysis is `rel_ancom`. At present this function is aimed at detecting differentially abundant taxa in two or more populations.

Input data according to the format used in the sample data files provided along with the R script. Specifically, one needs to provide two .csv files. The first containing the OTU counts data where one column is the "Sample.ID" and the all other columns are OTU counts corresponding to each id in the Sample ID column. The second file is the metadata file, where, again one column is the "Sample.ID" and the others contain various variables corresponding to each id, including the experimental group/population the id belongs to. For illustration purposes we are providing the publicly available microbiome data of Yatsunenko et al. (2012).

The function `rel_ancom` defined in the file "rel_ancom.R"" relies on a series of internal functions `normalizer_gm`, `normalizer_ref`, `pop_detect`, `reset_dat_missing` and `struc_zero` defined in the files, `geom_ref.R`, `pop_detect.R`, `stepA2.R` and `step A1.R` respectively. All these files, along with the sample data "otu.csv" and "meta.csv" should be stored in the working directory before proceeding. The analysis can be then be implemented as follows.

Input arguments:

1. `OTUdat`: The data set containing the otu counts of microbes along with a sample identifier column marked "Sample.ID"
2. `Vardat`: Date set containing the meta data. This can be a file containing all auxillary information, including the column containing the information on the main population of interest. As before, this file should also contain a sample identifier column marked "Sample.ID"
3. `pr`: a user chosen parameter controlling the mechanism for removal of structural zeros (see manuscript for details). Default `pr=0.05`
4. `pii`: a user chosen parameter controlling the mechanism for removal of outlier zeros (see manuscript for details). Default `pii=0.25`
5. `ref_name`: this argument controlls the reference function used to normalize data. (Default: logarithm of geometric mean accross each population level). (see manuscript for details)

Examples:

```
############################REQUIRED LIBRARIES######################################
library(dplyr)
######################READ DATA####################################################
otu <- read.csv("otu.csv", dec=",")
meta <- read.csv("meta.csv")
#####################################################################################
source("rel_ancom.R")
# Example 1. using logarithm of geometric mean as normalizing quantity.
rel_ancom(OTUdat=otu, Vardat=meta,main.var="country")
# Example 2. using logarithm of "p__Actinobacteria.g__Bifidobacterium" as normalizing quantity.
```

```r
rel_ancom(OTUdat=otu, Vardat=meta,main.var="country",
              ref_name="p__Actinobacteria.g__Bifidobacterium" )
```

Output: The output of the function `rel_ancom` is a list of two data frames.

1. `$detected_microbes`: a list of all microbes that are detected as being differentially abundant across the population of interest.
2. `$structural_zeros`: an array with the first column being the list of all microbes and the rest of the columns representing whether the corresponding microbe is structurally zero in a given population level. ("0" represents that a microbe is structurally absent in the population level).

A truncated output of Example 1 is provided in the following.

```
$detected_microbes
  [1] "p__mixed.g__"                           "p__Firmicutes.g__Acetivibrio"
  [3] "p__Proteobacteria.g__Achromobacter"     "p__Firmicutes.g__Acidaminococcus"
  [5] "p__Proteobacteria.g__Acidovorax"        "p__Proteobacteria.g__Acinetobacter"
  [7] "p__Proteobacteria.g__Actinobacillus"    "p__Actinobacteria.g__Actinomyces"
  [9] "p__Actinobacteria.g__Adlercreutzia"     "p__Firmicutes.g__Aerococcus"
 [11] "p__Proteobacteria.g__Aggregatibacter"   "p__Verrucomicrobia.g__Akkermansia"
 [13] "p__Bacteroidetes.g__Alistipes"          "p__Proteobacteria.g__Alkalimonas"
 [15] "p__Actinobacteria.g__Alloscardovia"     "p__Firmicutes.g__Anaerobacillus"
 [17] "p__Firmicutes.g__Anaerococcus"          "p__Firmicutes.g__Anaerofustis"
 [19] "p__Firmicutes.g__Anaerostipes"          "p__Firmicutes.g__Anaerotruncus"
 [21] "p__Firmicutes.g__Anaerovorax"           "p__Proteobacteria.g__Aquamonas"
 [23] "p__Actinobacteria.g__Arcanobacterium"   "p__Actinobacteria.g__Arthrobacter"
 [25] "p__Tenericutes.g__Asteroleplasma"       "p__Actinobacteria.g__Atopobium"
 [27] "p__Firmicutes.g__Bacillus"              "p__Bacteroidetes.g__Bacteroides"
 [29] "p__Actinobacteria.g__Bifidobacterium"   "p__Proteobacteria.g__Bilophila"
 [31] "p__Firmicutes.g__Blautia"               "p__Proteobacteria.g__Brachymonas"
 [33] "p__Spirochaetes.g__Brachyspira"         "p__Proteobacteria.g__Brenneria"
 [35] "p__Firmicutes.g__Brevibacillus"         "p__Tenericutes.g__Bulleidia"
 [37] "p__Proteobacteria.g__Burkholderia"      "p__Proteobacteria.g__Buttiauxella"
 [39] "p__Firmicutes.g__Butyrivibrio"          "p__Proteobacteria.g__Campylobacter"
 #######################TRUNCATED#############################################

$structural_zeros
                                           Microbe US VEN MA
1                                     p__mixed.g__  1   1  1
2                         p__Planctomycetes.g__A17  0   0  0
3                       p__Firmicutes.g__Abiotrophia  1   1  1
4                       p__Firmicutes.g__Acetivibrio  1   1  1
5                  p__Proteobacteria.g__Acetobacter  0   1  0
6                    p__Firmicutes.g__Acetobacterium  0   0  0
7                  p__Proteobacteria.g__Achromatium  0   1  0
8                p__Proteobacteria.g__Achromobacter  1   1  0
9                  p__Firmicutes.g__Acidaminococcus  1   1  1
10                p__Proteobacteria.g__Acidiphilium  0   0  0
11                   p__Proteobacteria.g__Acidisoma  0   0  0
12                  p__Proteobacteria.g__Acidomonas  0   0  0
13                  p__Proteobacteria.g__Acidovorax  1   1  1
14               p__Proteobacteria.g__Acinetobacter  1   1  1
15             p__Actinobacteria.g__Actinoallomurus  0   0  0
16           p__Actinobacteria.g__Actinoalloteichus  0   0  0
17              p__Proteobacteria.g__Actinobacillus  1   1  1
```

```
18            p__Actinobacteria.g__Actinobaculum  0   0  0
19           p__Actinobacteria.g__Actinocorallia  0   0  0
20         p__Actinobacteria.g__Actinokineospora  0   0  0
21             p__Actinobacteria.g__Actinomadura  0   0  1
22              p__Actinobacteria.g__Actinomyces  1   1  1
23        p__Actinobacteria.g__Actinomycetospora  0   1  0
24             p__Actinobacteria.g__Actinoplanes  0   1  0
25              p__Actinobacteria.g__Actinotalea  0   0  0
26             p__Bacteroidetes.g__Adhaeribacter  0   0  0
27            p__Actinobacteria.g__Adlercreutzia  1   1  1
28                  p__Firmicutes.g__Aerococcus  1   1  1
29            p__Actinobacteria.g__Aeromicrobium  0   1  0
30                p__Proteobacteria.g__Aeromonas  1   1  1
31                  p__Proteobacteria.g__Afipia  0   0  0
32          p__Proteobacteria.g__Aggregatibacter  1   1  1
33            p__Proteobacteria.g__Agrobacterium  1   1  0
34               p__Actinobacteria.g__Agrococcus  0   0  0
35                p__Actinobacteria.g__Agromyces  0   1  0
36             p__Verrucomicrobia.g__Akkermansia  1   1  1
37              p__Proteobacteria.g__Alcaligenes  0   0  0
38              p__Proteobacteria.g__Alcanivorax  0   0  0
39              p__Bacteroidetes.g__Algoriphagus  0   1  0
40           p__Proteobacteria.g__Alicycliphilus  0   1  0
######################TRUNCATED##############################################
```

Note: When supplying a user defined `ref_name` argument, it should be taken care that this reference microbe is non-zero in in all observations. If no such microbe exists, it is recommended to use the default setting using the geometric mean. Forcing a reference group with zero entries may return a solution which may not be reliable or the function may return an error.

Please contact Abhishek Kaul (akaul@math.wsu.edu) with any questions or concerns regarding the software
